# Interleukin-7 and Immunosenescence

**DOI:** 10.1155/2017/4807853

**Published:** 2017-04-06

**Authors:** Vanloan Nguyen, Andrew Mendelsohn, James W. Larrick

**Affiliations:** Panorama Research Institute, 1230 Bordeaux Drive, Sunnyvale, CA 94089, USA

## Abstract

The age of an individual is an important, independent risk factor for many of the most common diseases afflicting modern societies. Interleukin-7 (IL-7) plays a central, critical role in the homeostasis of the immune system. Recent studies support a critical role for IL-7 in the maintenance of a vigorous healthspan. We describe the role of IL-7 and its receptor in immunosenescence, the aging of the immune system. An understanding of the role that IL-7 plays in aging may permit parsimonious preventative or therapeutic solutions for diverse conditions. Perhaps IL-7 might be used to “tune” the immune system to optimize human healthspan and longevity.

## 1. Introduction

One's chronological age is an important, independent risk factor for many of the most common diseases afflicting the aging population of the world. Thus, understanding the central mechanisms driving senescence may have global impact to improve healthspan and reduce healthcare costs. A diverse group of degenerative pathological processes contribute to the decline and dysfunction of multicellular organisms. Among these are fibrosis, calcification, stem cell drop-out, nonenzymatic glycation, degradation of extracellular matrix (ECM), compromised mitochondrial biogenesis, loss of tissue homeostasis, and uncontrolled inflammation. Immunosenescence is the lifelong reduction in immunological reserve and homeostasis. This process contributes to reduced resistance to infectious diseases (e.g., pneumonia, influenza, meningitis, and urinary tract infections), increased propensity to develop cancer, and increased autoimmune disease (e.g., rheumatoid arthritis, thyroiditis, systemic lupus erythematosus, and multiple sclerosis) observed in aged individuals. Furthermore, immunosenescence limits the success of medical interventions such as vaccination and efforts to augment antitumor immunity. Attempts to pinpoint a single “cause” of senescence in general and immunosenescence in particular have met with limited success. However, recent studies support a critical role for IL-7 in the maintenance of a vigorous healthspan and have identified IL-7 and its receptor and associated proteins, “the IL-7 network,” as a useful biomarker of successful aging [1].

To understand the IL-7 network, we begin with a description of IL-7, the IL-7 receptor, and downstream signal transduction. We document how aging affects various parts of the immune system, B cells, T cells, and so forth, in an effort to understand which aspects of the elegant immune mechanism are most vulnerable and connected to IL-7. Next, we examine mechanisms of immunosenescence through the prism of the “molecular and cellular hallmarks of aging” as defined by Lopez-Otin et al. [2]. Among these hallmarks are as follows: (a) increasing damage to DNA, (b) genomic instability and epigenetic changes, (c) telomere shortening, (d) stem cell exhaustion, (e) limited capacity for regeneration, (f) loss of proteostasis, (g) senescence of cells (e.g., Hayflick limit), and (h) altered communication between cells, tissues, and organs. Each of these mechanisms (and probably more!) contributes to the development of immunosenescence. Finally, we describe efforts to utilize the IL-7 axis for therapeutic purposes. While initial attempts to develop therapeutics based on the IL-7 network have met with limited success, efforts are ongoing to harness the pleiotropic activities of this “lympho-homeostatic” cytokine.

## 2. IL-7, IL-7 Receptor, and IL-7 Signal Transduction

**Figure 1:**
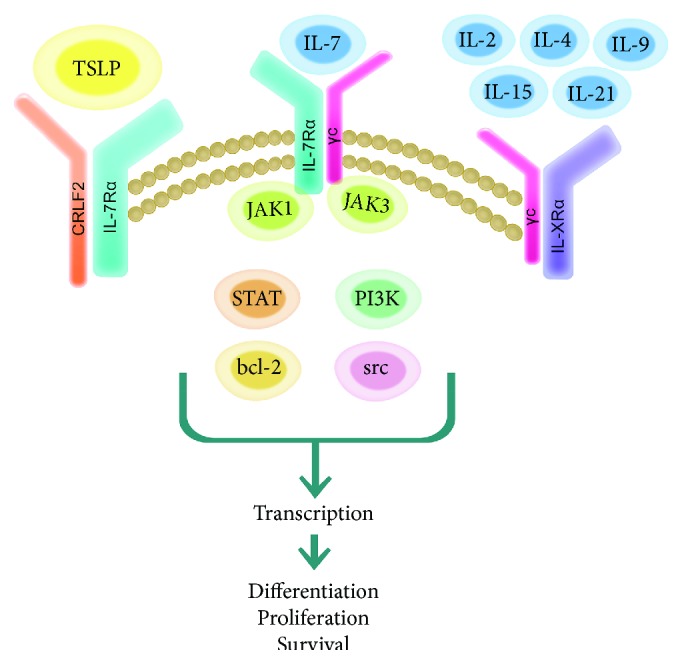
Schematic of IL-7R signal transduction. IL-7 and its cell-surface receptor—a heterodimer consisting of the IL-7R*α* and the common *γ* chain (*γ*c)—form a ternary complex that engages the JAK-STAT pathway. The subsequent downstream activation of PI3K, bcl-2, and src kinases leads to gene transcription. IL-7R signal transduction is important in directing the differentiation, proliferation, and survival of immune cells including B, T, and natural killer (NK) cells. The IL-7R*α* chain is shared with another receptor recognizing thymic stromal lymphopoietin (TSLP). In this scenario, the IL-7R*α* noncovalently associates with the cytokine receptor-like factor 2 (CRLF2). Likewise, the *γ*c is shared with other receptors specifically recognizing IL-2, IL-4, IL-9, IL-15, and IL-21.

IL-7 is a member of the common *γ* chain (*γ*c-CD132) family of cytokines that include interleukin-2 (IL-2), IL-4, IL-7, IL-9, IL-15, and IL-21 [3, 4]. Like other members, IL-7 signals via a ternary complex formed with its unique *α*-receptor, IL-7R*α* (CD127), and the common *γ*c receptor (Figure 1). This interaction stimulates the Janus kinase (JAK) and signal transducer and activator of transcription (STAT) proteins with subsequent activation of the phosphoinositol 3-kinase (PI3K)/Akt, or Src pathways to facilitate target gene transcription [3].

Two forms of the IL-7R, membrane-bound and soluble IL-7R, exhibit different functions [5, 6]. Membrane-bound IL-7R mediates IL-7 signal transduction, while soluble IL-7R provides a modulatory control mechanism [7]. Lundstrom et al. (2013) suggest that the soluble form of the IL-7R may potentiate IL-7 signaling and augment autoimmunity [8].

The signaling cascade(s) initiated by *γ*c interleukins and their receptors regulates homeostasis of B, T, and natural killer (NK) cells of the immune system.

## 3. Immunosenescence: The Result of Multiple “Hits” to Immune Cell Development

Immunosenescence affects multiple cells within the hematopoietic lineage as described below. The result is a gradual deterioration of immune function with age. Disruption of the IL-7 signaling pathway plays a central role in this process.

### 3.1. Hematopoietic Stem Cells

**Figure 2:**
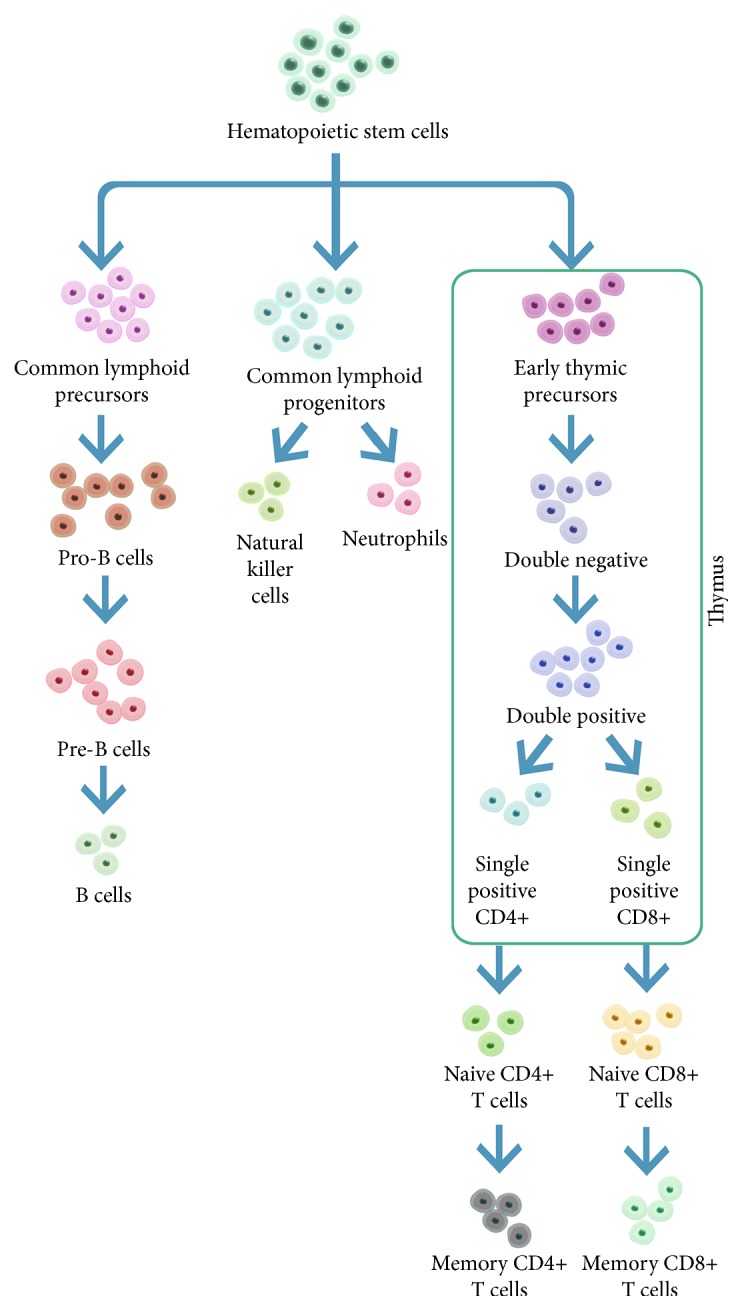
The development of lymphocytes. CLP cells are the earliest lymphoid progenitor cells derived from hematopoietic stem cells, giving rise to T-lineage, B-lineage, and natural killer (NK) cells. lL-7 plays a significant role at specific stages in the development of these cells. (See text for details.)

The diverse cells of the immune system derive from bone marrow-derived hematopoietic stem cells (HSC) (Figure 2). HSC self-renew to prevent clonal exhaustion and differentiate along multiple lineages. Aging HSC demonstrate a reduced capacity for self-renewal [9, 10] and ability to differentiate along the lymphoid lineage [11] which is accompanied by an age-dependent increase in cell-cycle inhibitor p16^INK4a^ [11].

### 3.2. B Lymphopoiesis

**Figure 3:**
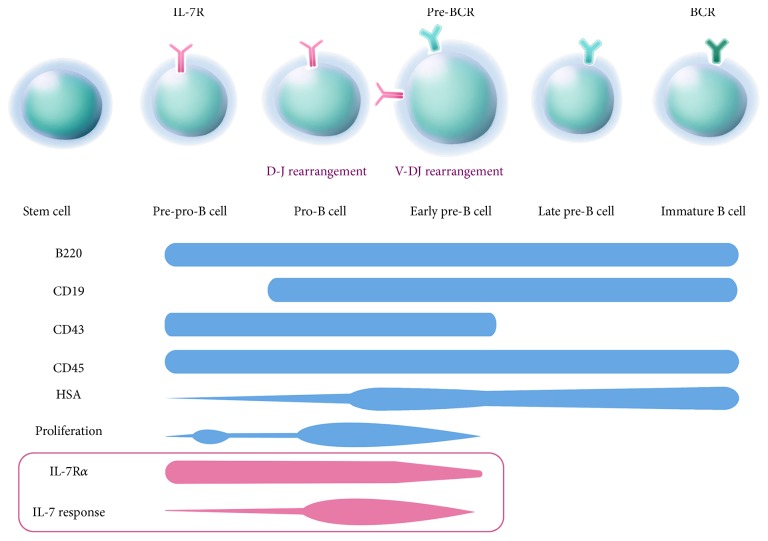
Schematic of B lymphopoiesis in the bone marrow. The presence of various cell-surface markers is indicated in parallel with each stage in the development of B cells. Among these, IL-7 is important in early B cell differentiation because it promotes the commitment of CLP to the B-lineage. It also acts in concert with transcription factors to regulate immunoglobulin gene rearrangement in the pro-B cell and early pre-B cell stages. Early pre-B cells express IL-7R*α* until V-DJ rearrangement is complete. Successfully rearranged cells then proliferate in response to IL-7 and other cytokines.

Antibody-producing B cells arise in the bone marrow from HSC via the common lymphoid precursor (CLP) cell [12] (Figure 3). CLP cells give rise to early pro-B cells (CD43+, CD45+, and MHCII+) via successful D-J heavy chain gene joining in an IL-7 receptor-dependent manner. The activity of the recombination activating genes (RAG1/RAG2) controls rearrangement and assembly of immunoglobulins [13]. Late pro-B cells (CD43+, CD45+, CD40+, CD19+, and MHCII+) undergo heavy chain VH joining to the DJ fragments [14]. Next large pre-B cells express the pre-B cell receptor complex (BCR: surface immunoglobulin, +CD79a/b coreceptor) and differentiate into small pre-B cells characterized by light chain DJ rearrangement.

Bone marrow development of B cells is characterized by both positive and negative selection. Cytokines such as B cell-activating factor (BAFF, tumor necrosis factor ligand superfamily member 13B), a proliferation-inducing ligand (APRIL, tumor necrosis factor ligand superfamily member 13 (TNFSF13)), and engagement of the BCR drive B cell expansion. If the BCR does not bind a ligand, the further development of the B cell is interrupted and the cell undergoes apoptosis (positive selection). If the BCR binds a self-antigen, central tolerance can result via several mechanisms, that is, clonal deletion, receptor editing, or anergy [15]. To complete development, the IgM-bearing immature B cells exit the bone marrow circulating to peripheral lymphoid tissues such as the spleen. There, B cells can undergo further activation, affinity maturation, and immunoglobulin class-switching eventually differentiating into antibody-producing plasma cells.

IL-7 plays multiple important roles during B cell lymphopoiesis. Prior to very early B cell development, IL-7 promotes the commitment of CLP cells to the B-lineage [16]. Pro-B cells bear IL-7 receptors that facilitate the survival, proliferation, and maturation to pre-B cells. IL-7 acts in concert with Pax-5, E2A, and EBF and other transcription factors to regulate immunoglobulin gene rearrangement by modulating Foxo protein activation and RAG enhancer activity. Similar to other cytokines, IL-7R signaling is regulated by the suppressor of cytokine signaling (SOCS) proteins [17].

Age-associated changes in humoral immunity include reduced numbers of pro- and pre-B cells with diminished RAG expression [18, 19] resulting from a compromised bone marrow microenvironment [20]. These defects in B cell development result in the generation of less effective antibodies with reduced diversity and lower affinity [21, 22] as well as production of autoantibodies, a result of compromised tolerance. Murine pro-B cells fail to respond to IL-7 but not to other growth factors [23], a finding consistent with an important role of IL-7 in aging of the hematopoietic microenvironment.

### 3.3. T Lymphocytes and Thymic Involution

**Figure 4:**
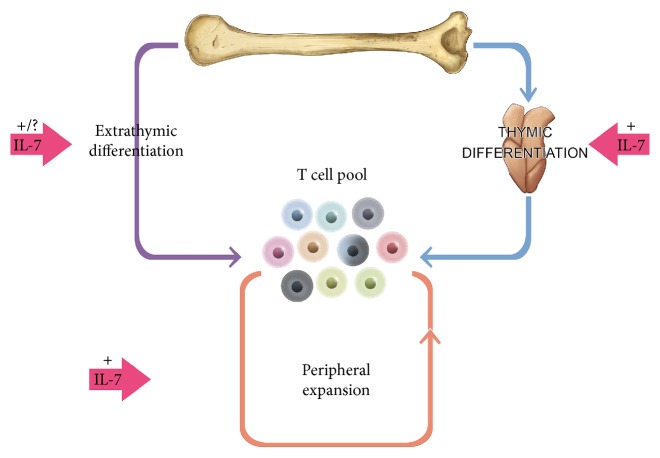
Effect of IL-7 on T cell regeneration. IL-7 regulates T cell homeostasis through three immune modulation pathways: thymic differentiation, peripheral expansion, and extrathymic differentiation. To regenerate peripheral T cells, IL-7 directs T cell differentiation and maturation in the thymus. As age-related decline in thymic function becomes apparent, IL-7 contributes to the maintenance of the T cell pool through the expansion of existing peripheral T cells. Extrathymic differentiation from CLP cells is possible but is only a minor pathway. In all three scenarios, IL-7 is known to have an important signaling effect.

The thymus plays a central role in immunity being the primary organ facilitating T cell maturation and development of self-tolerant, self-major histocompatibility complex-restricted, immunocompetent T cells [24–26] (Figure 4). A dramatic reduction in the size of the thymus accompanies aging suggesting a critical role in immunosenescence. Thymic epithelial cells (TEC) provide a favorable stromal microenvironment to nurture T cell development [27, 28]. Paracrine growth factors such as IL-7, other cytokines, and cell-surface antigens combine to direct thymopoiesis. The process begins when HSC-derived early thymic precursor (ETP) cells that are surface negative for CD4 and CD8 (so-called “double negative cells”) traffic into the thymus via the corticomedullary junction and localize in the outmost cortical zone. These double negative ETP cells develop along the following pathways; DN1 [CD44+ CD25−], DN2 [CD44+ CD25+], DN3 [CD44− CD25+], and DN4 [CD44− CD25−]. Most cortical thymocytes next express CD4 and CD8 (becoming double positive) during rearrangement of T cell receptor (TCR) genes [29, 30] and selection against self-reactive antigens. Finally, migration to the thymic medulla is accompanied by differentiation into single-positive CD4+ or CD8+ cells ready to populate peripheral lymphoid tissues [31].

The so-called “thymic involution” is characterized by reduced epithelial space and cellularity accompanied by an increase in perivascular space and a progressive replacement with fat [32–34]. This process begins as early as age 9 months [35] with several phases of more rapid regression (in those under 10 years of age and between the ages of 25 and 40 years) with slower atrophy thereafter [33]. Despite these dramatic changes, the aging thymus continues to produce T cells albeit at a reduced rate [36, 37]. For example, Jamieson et al. (1999) found evidence of recent thymic emigrants (RTE) among elderly individuals by documenting T cell receptor excision circle-positive (TREC+) T cells [37].

Numerous studies have tried to nail down the cause of thymic aging. IL-7 is a strong candidate because it is produced by TECs and is critically important for thymopoiesis. IL-7 declines with age, and treatment of mice with anti-IL-7 antibodies elicits a phenotype similar to thymic involution [38]. In some studies, IL-7 therapy of old mice increases thymic cellularity and weight. However, Sempowski et al. demonstrated an increase in peripheral TREC+ CD8+ T cells following treatment with IL-7 for two weeks, although this therapy did not increase the number of thymocytes [39]. When Phillips et al. engineered thymic stromal cells to produce exogenous IL-7, older mice displayed increased CD25+ DN thymocytes but exhibited no change in thymic output or reversal of thymic involution [40]. Apparently, IL-7 can reverse a checkpoint defect early in thymopoiesis without benefit for reversal of thymic involution.

**Figure 5:**
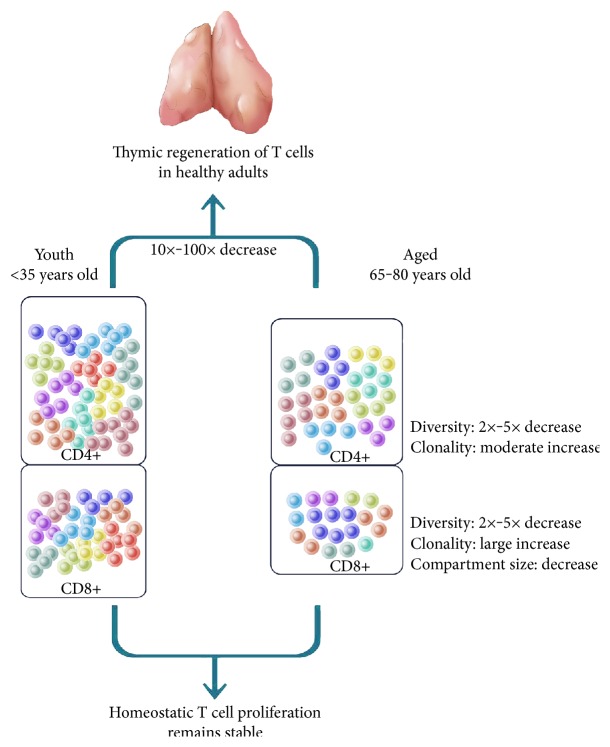
While thymic regeneration of T cells in healthy adults experiences a 10–100-fold decrease, homeostatic T cell proliferation remains stable throughout life. Both T cell lineages decrease by 2–5-fold in diversity. However, differences in the homeostatic proliferation of CD4+ and CD8+ cells drive the imbalance of these cells observed in aging: there is a modest increase in the clonality of CD4+ cells but a large increase for CD8+ cells with a concomitant decline in the size of the CD8+ compartment.

The generation of thymic T cells decreases by perhaps 100 times with age. Although the generation of novel naive T cells is reduced, the diversity of the youthful T cell repertoire is maintained during aging by homeostatic proliferation (Figure 5). Overall, the richness of the repertoire is only reduced by perhaps 2–5-fold for both CD4+ and CD8+ lymphocytes. However, differences in the homeostatic proliferation of CD4+ and CD8+ cells drive the imbalance of these cells observed in aging: there is a modest increase in the clonality of CD4+ cells but a large increase for CD8+ cells with a concomitant decline in the size of the CD8+ compartment [41, 42].

IL-7 drives T cell proliferation without inducing a switch to a memory phenotype [43]. Concentrations of IL-7 do not decline with age and thus are unlikely to become rate limiting in the peripheral expansion of naive T cells which is driven by tonic TCR signals and IL-7 [44, 45]. This is consistent with the observations that the CD4+ lymphocyte compartment is not reduced in size with aging. Naive CD4+ T cells from aged individuals express more high-affinity IL-2 receptors (CD25) becoming more IL-2 responsive [46] which undoubtedly supports their survival. CD31 is expressed on a subset of naive CD4+ T cells that are recent thymic emigrants. TREC concentrations decrease slightly in CD31+ CD4+ T cells with age consistent with only limited replication in this population [47]. With thymic involution, the CD31+ population shrinks and the CD31−naive T cell population increases in size and its T cell receptor excision circle content more drastically declines [47]. The loss of CD31 expression in naive T cells is generally not associated with the acquisition of phenotypic markers of memory T cells. Clones derived from the naive CD8+ T cell compartment largely maintain a naive phenotype as only a small fraction of these clones expresses memory cell markers. Furthermore, TCR diversity in CD8+ memory T cells is about tenfold lower than that of CD4+ memory T cells [48]. Presently, the reasons for the large depletion of the CD8+ compartment are not clear.

## 4. IL-7R Network as a Biomarker of Aging

The age of an individual is an important, independent risk factor for many of the most common diseases afflicting modern societies. Biomarkers can improve our understanding of age-associated conditions, changes in status of health, morbidity, and mortality by documenting biological age versus chronological age [1]. Recent studies out of Netherlands indicate that IL-7 and its receptor may provide promising biomarkers of immunosenescence.

As part of Netherlands' Leiden Longevity Study (LLS), Passtoors et al. (2015) identified a set of 1853 genes that associated with chronological age. 244 of this panel were associated with increased familial longevity, that is, they were differentially expressed in middle-aged children of siblings of healthy nonagenarians [1]. Among these genes, low expression of the IL-7 receptor (IL-7R) in old age was associated with a *better* metabolic profile (e.g., insulin sensitivity and lower blood lipids) and thus nominated as a candidate biomarker for healthy aging [49]. This does not imply that reduction of IL-7R expression in general is beneficial to maintaining good health. Proteins interacting with the IL-7R were identified using the STRING protein-protein interaction database (http://string-db.org/). This approach identified the “IL-7R network” which is comprised of the following proteins: IL2RG, IL-7, TSLP, CRLF2, JAK1, and JAK3. A short description of their function(s) demonstrates how they are connected.

### 4.1. IL-7 and IL-7R

IL-7 and its receptor, IL-7R/CD127, regulate development, differentiation, and survival of T cells at multiple levels. The Leiden Longevity Study group previously observed that the offspring of nonagenarian siblings do not exhibit the expected age-related reduction of peripheral naive T cells [50]. Reduced IL-7 signaling may somehow protect naive T cells with benefit to the biological age and health status of elderly individuals. A more robust naive T cell repertoire is expected to provide better protection from novel pathogens encountered as one ages.

Levels of gene expression of the IL-7R gene network were measured by RT-PCR of whole blood samples from 87 nonagenarians who also had a nonagenarian sibling, 337 of their offspring, considered “healthy agers,” and 321 controls from the LLS. After statistical adjustment for multiple group testing, three genes, IL-7R, IL2RG, and IL-7, showed significant differential expression with >5% difference between nonagenarians and middle-aged controls. Expression of the ligand genes of the IL-7R complex (i.e., IL-7R, IL2RG, IL-7, TSLP, and CRLF2) was all *lower* in nonagenarians, whereas expression of JAK1 and JAK3 was higher. The expression level of the IL-7R gene set was significantly different between the nonagenarians and younger controls (*P* = 4.6 × 10^−4^). Among the offspring of the nonagenarians, expression of the IL-7R complex/ligands IL-7R, IL2RG, IL-7, TSLP, and JAK3 was *lower* versus that of the controls with the IL-7R exhibiting the most significant difference. In contrast, JAK1 was higher among offspring. However, higher expression of IL7R is actually associated with protection from mortality (see below). How does one explain this paradox?

### 4.2. Interleukin-2 Receptor Gamma

Interleukin-2 receptor subunit gamma (IL2RG), also called the common gamma chain (*γ*c), or CD132, is an essential component of interleukin signaling involving IL-7, IL-2, IL-4, IL-9, IL-15, and IL-21. CD132 is a 369 amino acid glycoprotein that participates in the initiation of signaling cascades that direct the differentiation and maturation of lymphocytes, including T cells, B cells, and NK cells. CD132 is encoded by the gene IL2RG, located on the q arm of the X chromosome at position 13.1. X-linked SCID is caused by one of over 200 mutations in this gene. For reasons that are unclear, overexpression of the gene may impair the immune response in neuropsychiatric disorders, including schizophrenia. Pathways involving IL2RG are involved in human T cell lymphotropic virus type 1 (HTLV-I) infection and the T helper 17 cell (Th17) differentiation pathway [51].

### 4.3. Thymic Stromal Lymphopoietin (TSLP)

Thymic stromal lymphopoietin (TSLP), a member of the cytokine I receptor family, binds heterodimeric CRLF2 and the IL-7R*α* and activates the STAT3, STAT5, and JAK2 pathways. These pathways control differentiation and proliferation of hematopoietic stem cells. Fibroblasts, epithelial cells, and stromal cells all express TSLP. TSLP production is stimulated by inflammatory cytokines such as IL13, by pathogens or physical injury [52]. Triggering toll-like receptor 3 (TLR-3) can also induce TSLP secretion. Elevated expression of dendritic cell expressed TSLP contributes to the pathogenesis of asthma [53], atopic dermatitis [54], and inflammatory arthritis [55], diseases known to be associated with abnormal regulation of IL-7.

### 4.4. CRLF2

Cytokine receptor-like factor 2, a member of the cytokine I receptor family, is a receptor for TSLP. Heterodimerization of CRLF2 with the IL-7R*α* by TSLP initiates JAK-STAT phosphorylation-mediated transcriptional control as noted above.

### 4.5. JAK1 and JAK3

Cytokine signaling crosstalk results from cross-specificity in activation by the Janus kinase (JAK) and signal transducers and activators of transcription (STAT). The JAK family comprises four members, each associated with particular cytokine signal transduction. The JAK-STAT pathway is initiated following binding of IL-7 to its receptor complex. Subsequently, phosphorylation of tyrosine residues on the receptor takes place, thus recruiting STATs, which in turn are tyrosine-phosphorylated by JAKs. The activated STATs dimerize and migrate to the nucleus to induce transcription of targeted genes. Stimulation of numerous STATs by the JAK kinases leads to significant cross-talk. Research indicates that clathrin phosphorylation is caused by the activation of the pro-T cells by IL-7. Severe combined immunodeficiency (SCID) syndrome results from aberrant JAK signaling [56]. Mutation in the JAK3 kinase gene causes autosomal SCID with increased susceptibility to life-threatening opportunistic infections during infancy.

### 4.6. Mortality and IL-7R Gene Expression

In the Leiden Longevity Study, survival analysis was made using a Cox proportional hazard model for low versus high IL-7R gene expression in 81 nonagenarians versus the combined group of 619 of their middle-aged offspring and controls. Among nonagenarians (hazard ratio (HR) = 0.60, 95% CI 0.40–0.92, *P* = 0.018) as well as middle-aged subjects (HR = 0.50, 95% CI 0.30–0.82, *P* = 0.007), high IL-7R gene expression is associated with *reduced mortality over 10 years*; that is, higher gene expression levels of IL-7R in blood predict better survival in both age groups. Seemingly, high levels of IL-7R are beneficial. Do the middle-aged children of nonagenarians also have higher mortality over a 10-year period? The data do not have the statistical power to answer this question. But assuming the children follow the pattern of the entire middle-aged group, they may be more resistant to immunosenescence at a cost of poorer short-term health/immune function and an increased chance of death over the short term. It is as if there is a limited total supply of lymphocytes that can be induced by IL-7 over a lifetime. Consuming the lymphocytes in youth and middle age provides better health, with the caveat that it may limit the possibility of living to old age. Fewer/less active lymphocytes during middle age may increase the chance of disease somewhat but result in a large enough pool of lymphocytes in old age to promote viability. Perhaps IL-7R represents a case antagonistic pleiotropy.

Alternatively, the authors speculate that perhaps the nonagenarian members of long-lived families exhibit more “efficient” IL-7 signaling, requiring fewer naive T cells to preserve their immunological functional reserve to combat infections in old age. This contrasts with individuals with severe combined immunodeficiencies (SCIDs) and HIV infections who exhibit very low IL-7R signaling [57–59].

### 4.7. Higher IL-7R Expression Is Associated with a Higher Prevalence of Immune-Related Diseases

Interestingly, in the LLS, the difference in IL-7R expression between offspring and controls remained after adjustment for prevalence of T2D, COPD, and RA. Thus, the IL-7R expression levels in blood associated with immune-related disease on the one hand and decreased mortality over a 10-year period on the other, suggesting that increased immune health may trade off with an increased chance of autoimmune and inflammatory diseases.

## 5. Paradigms and Mechanisms of Immunosenescence

Lopez-Otin et al. (2013) recently summarized current “Molecular and cellular hallmarks of aging” [2]. Among these were increasing damage to DNA, genomic instability and epigenetic changes, telomere shortening, stem cell exhaustion, limited capacity for regeneration, loss of proteostasis, senescence of cells (e.g., Hayflick limit), and altered communication between cells, tissues, and organs. All of these mechanisms (and probably more!) contribute to the development of immunosenescence.

### 5.1. Damage to DNA

Many syndromes of premature aging are characterized by defects in DNA repair pathways [60, 61]. Accelerated immune system aging has been linked to defects in the MRE-ATM DNA repair pathway in patients with rheumatoid arthritis which renders naive CD4 T cells more susceptible to apoptosis [62]. Similar damage gradually accumulates in aged individuals, although damage to DNA in naive CD4 T cells in normal individuals is minimal up to the 7th decade of life [63]. Perhaps as important, accumulation of damage to mitochondrial DNA (mtDNA) of lymphocytes is well documented and is likely to play an important role in immunosenescence [64, 65].

### 5.2. Integrity of Telomeres

The termini of chromosomes are comprised of repetitive nucleotide sequences called telomeres. The average human telomere encodes ~2500 copies of the hexamer, TTAGGG. Over the lifetime of an individual, telomeres shorten with each cell division, beginning from about 11 kilobases at birth to less than 4 kilobases in old age. The telomerase reverse transcriptase (TERT) gene and associated telomerase RNA component (TERC) as RNA template catalyze the addition of the telomeric repeats to the chromosome ends to prevent shortening in germ-line cells, embryonic stem cells, and the majority of tumor cells.

Telomerase activity and telomere length correlate with stage of differentiation and activation of B and T lymphocytes [66]. Age-associated telomere attrition in naive T cells is well documented despite the continued expression of telomerase [67]. Memory T lymphocytes and naive T lymphocytes differ in their telomeric lengths and replicative capacity [68]. Defects in proliferation of effector memory T cells correlate with markers of senescence such as CD57 and shorter telomere length [69]. Brazvan et al. (2016) showed that among a panel of cytokines, IL-7 and IL-15 increased telomere length and hTERT gene expression in cord blood cells consistent with the function of these cytokines to maintain the naive phenotype of progenitor cells [70]. Although telomere dysfunction accompanies aging and autoimmunity (e.g., rheumatoid arthritis [63]), “telomere-based” therapies for immunosenescence [71, 72] have to date largely failed.

### 5.3. Metabolic Mechanisms of Cellular Aging

Telomere attrition, DNA damage responses, mtDNA damage, and mitochondrial dysfunction are intimately connected to the metabolic changes associated with aging [73, 74]. Like tumor cells, aerobic glycolysis enables T cell proliferation [75]. Mitochondria-derived reactive oxygen species (ROS) play a key role in T cell receptor-mediated signaling [76]. Autophagy provides another level of control of T cell differentiation and survival [77, 78], and in mice, aging of T cells may be associated with a defect in chaperone-mediated autophagy [78].

mTOR signaling has been implicated in mammalian lifespan and human longevity [79, 80]. AMPK [81], a sensor of low intracellular glucose, is activated by phosphorylation on Thr172 in its catalytic *α*-subunit to shift metabolism towards catabolism and inhibit mTOR activity in T cells [82, 83]. The senescent CD27-CD28-CD4+ T cell subset showed spontaneous AMPK*α* activation and downstream p38 signaling [84]. In senescent CD8+ T cells, p38 signaling inhibited mTORC1-independent autophagy.

IL-7 augments phosphorylation of S6 and 4EBP1 which are downstream targets of the mTOR complex 1 (mTORC1). Rapamycin, known to extend the lifespan of several species including mice, blocks these effects of IL-7. The stimulation of pre-B leukemia cells by IL-7-R complex member TSLP is also inhibited by rapamycin [85].

Ouyang et al. (2009) implicated IL-7R as a Foxo1 target gene [86], and Kerdiles et al. (2009) showed that Foxo1 links homing and survival of naive T cells by regulating L-selectin, CCR7, and the IL-7R [87]. Furthermore, IL-7 induces Foxo1 phosphorylation with resultant target gene transcription [87]. In B cells, the mTOR complex 2 (mTORC2) suppresses IL-7R gene expression by regulating Foxo1 phosphorylation [88].

Reduced IL-7 levels may contribute to lower mTORC1 activation, and IL-7R signaling correlates with mTORC2 activation of RPTOR, Foxo1, and mTOR genes [1, 79]; both effects associated with increased lifespan/longevity. Higher mTOR levels reduce HSC function [89], which could result in fewer lymphocytes later in life. Considering the clear connections with IL-7 signaling and similar findings on the level of gene expression variations, mTOR signaling might also be involved in “inflammaging” as part of its lifespan-regulating effect.

### 5.4. Inflammaging

**Figure 6:**
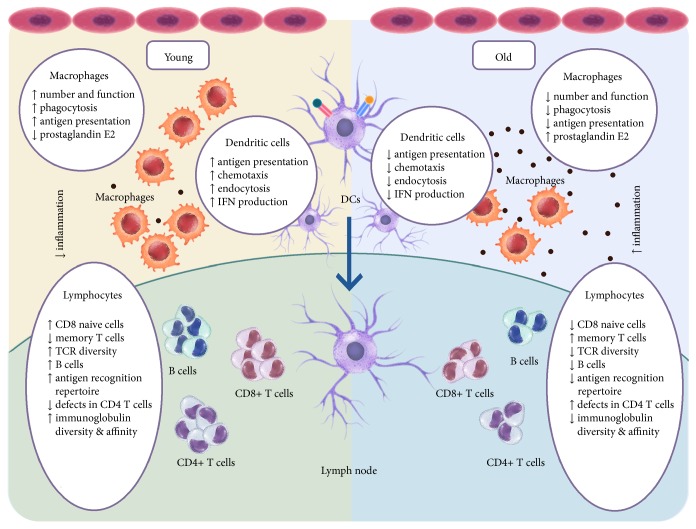
Inflammaging drives immunosenescence. Chronic low-grade inflammation results from reduced function of the aging innate and adaptive immune systems. In old age, the number of macrophages, as well as phagocytosis, decreases. Likewise, dendritic cells lag in antigen uptake, as well as in migration to lymph nodes. While B and T cell effector responses become less than optimum, a notable expansion of memory CD8+ T cells is observed.

The “inflammaging” hypothesis postulates that systemic inflammation makes a significant contribution to the process of aging [90, 91] (Figure 6). Healthy nonagenarians from the Leiden study display fewer age-related characteristics of “immunosenescence” and relatively low levels of proinflammatory markers [50]. The paradigm proposes that reduced function of the aging innate and adaptive immune systems promotes low-grade inflammation mediated by diverse mechanisms. Chronic infections (e.g., herpes simplex-1 virus (HSV-1) and cytomegalovirus (CMV)) and various endogenous antigens such as oxidized lipoproteins, advanced glycation end products, cellular debris, misfolded proteins and products of nitrosylation, reactive oxygen intermediates, and so forth stimulate the immune system. By interacting with danger-associated molecular pattern (DAMP), recognition molecules such as the toll-like receptor (TLR) chronic activators reduce effective responses to infections and cancer, while mediating low-grade tissue damage. Immunosenescence is characterized by the deterioration of immune function at multiple levels, often driven by chronic low-grade inflammation.

### 5.5. The Senescence-Associated Phenotype (SASP)

One postulated origin of proinflammatory cytokines may be the senescent cells themselves. The senescence-associated phenotype (SASP) characterizes old cells that have not been eliminated by the aging immune system. While cell senescence may reduce the odds of carcinogenic transformation, damaged cells with the cytokine secretory phenotype accumulate promoting tissue disruption, atrophy, and dysfunction. Recent remarkable studies demonstrate that clearance of p16^Ink4a^-positive senescent cells delays aging-associated disorders in mice [92].

A unique subset of B cells, age-associated B cells (ABC) expressing the transcription factor T-bet, accumulate progressively with age [93]. Aged mice exhibit ~5–10-fold increase in ABC with a higher proportion in females and autoimmunity. These cells promote apoptosis of pro-B cells via secretion of TNF*α* [94]. Whether the age-dependent reduction in IL-7-mediated pro-B to pre-B cell checkpoint transition relates to the activity of ABC will await future study.

### 5.6. Stem Cell Attrition versus Regeneration

In the adaptive immune system, the generation of novel naive T cells is completely dependent on thymic function (see 2.3). Thus, the dramatic involution of the mammalian thymus supports a key role in T cell homeostasis with aging. However, recent work documents substantial differences between short-lived rodents and much longer living humans [95]. In humans, maintenance of a robust repertoire during adulthood is dependent on homeostatic proliferation of the existing CD4 T lymphocyte pool versus repopulation from the thymus [96]. IL-7 is uniquely important in the homeostatic maintenance of and peripheral expansion of naive T cells [44, 45]. Concentrations of IL-7 do not decline with age and do not become limiting in this process. Thus, IL-7 augments naive CD4 T cell proliferation without promoting a switch to memory cells [43].

In contrast to CD4 cells, the CD8 lymphocyte compartment shrinks with age. The reasons for this are not clear [42, 48]. Furthermore, T cell receptor diversity is about tenfold lower for CD8 versus CD4 memory cells [97]. Reynolds et al. (2013) suggest that increased IL-7-mediated proliferation secondary to a reduced threshold for IL-7R activation may lead to a steep decline in lymphocyte populations with aging [98]. Thus, the balance between TCR engagement and homeostatic cytokine stimulation needs to be “tuned” for optimal survival/maintenance versus triggering increased turnover [99]. Production of excess cytokines and their receptors (e.g., IL-2 and interferon gamma) during inflammation is expected to upset this balance to accelerate immunosenescence. Thus, not unexpectedly, several autoimmunoinflammatory diseases are associated with accelerated immunosenescence [100].

## 6. Medical Conditions Associated with IL-7/IL-7R Dysfunction

Numerous studies implicate IL-7 and IL-7R signaling in the pathogenesis of immunoinflammatory disorders [101–104]. Meta-analysis and genome-wide association studies demonstrate that genetic variation in the IL-7R gene is associated with type 1 diabetes [105], multiple sclerosis [106–108], ulcerative colitis [109], and primary biliary cirrhosis [110]. These results support the hypothesis that genetic variation in and the expression of the IL-7R gene contributes to the pathogenesis of autoimmune and chronic inflammatory disease. However, many of these diseases are multigenic and the IL-7R network probably accounts for only a fraction of the variation.

Gain-of-function mutations as well as insertions and deletions in the IL-7R*α* gene frequently occur in both B and T cell acute lymphocyte leukemias (ALL) [52, 111–113]. Details of multiple structural insights into the aberrant IL-7 signaling observed in malignant leukemia cells are described in beautiful detail by Walsh [52].

Mutations in the extracellular domain of IL-7R*α* are associated with severe combined immunodeficiency (SCID) of the T− B+ NK+ type [57, 114]. Premature termination of the mRNA, folding defects, and/or destabilized IL-7-R*α*-receptor protein compromises interactions with IL-7 to limit signal transduction with global effects on immune function. More than 300 mutations have been identified in the *γ*c receptor in patients suffering from X-linked SCID (see http://research.nhgri.nih.gov/scid/). The phenotype of *γ*c SCIDs is T− B+ NK− [114]. Bone marrow transplantation is the current therapy for both IL-7R*α* and *γ*c SCIDs. Do SCIDs exhibit accelerated aging? This is an interesting, but probably impossible question to answer, given the complexity of these patients and their generally shorter average lifespans due to infectious complications.

### 6.1. Clinical Experience with IL-7

**Figure 7:**
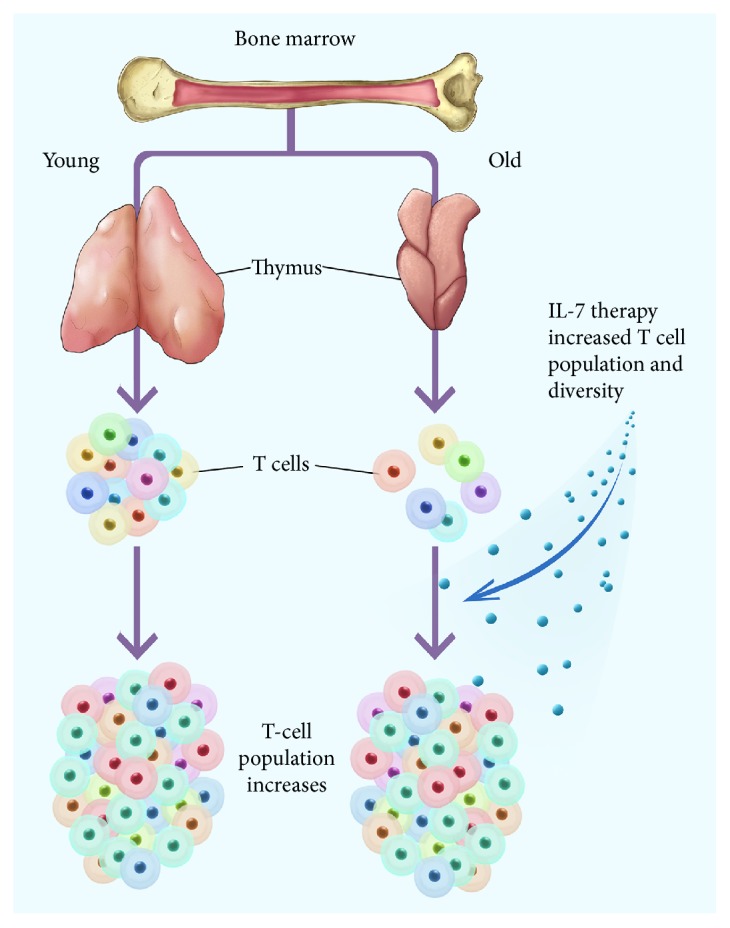
In some studies, Il-7 therapy caused rejuvenation of the T cell population, broadened immune responses in cancer patients, and enhanced T cell recovery in HIV-1-infected adults. (See Section 6.)

Recent studies have shown that (1) in treated HIV-infected adult patients [115, 116], thymic function can be maintained or improved to facilitate CD4 lymphocyte repopulation and (2) growth hormone treatment [111] or androgen antagonism [117, 118] can augment adult thymopoiesis. Thus, thymic atrophy may be at least partially reversible and/or immunosenescence can be delayed. Furthermore, the human adult thymus may have more reserve than previously thought [69, 119]. Based on its pleiotropic activities, replacement or augmentation of IL-7 is a rationale therapeutic approach to counteract immunosenescence (Figure 7).

### 6.2. Pre-Pro-B Cell Growth-Stimulating Factor (PPBSF)

Both IL-7 (reviewed in Sportes et al. [120]; Capitini et al. [121]) and HGF (reviewed in Ido and Hirohito [122]) have been tested in numerous human clinical trials as separate agents and, in general, have been well tolerated and mediated some beneficial effects. Administration of IL-7 to normal individuals caused an expansion of CD8+ and CD4+ T cells but a relative decrease in T-regulatory cells [123]. Also, in normal individuals, IL-7 increased the T cell receptor diversity by preferential expansion of naive T cell subsets resulting in a circulating T cell pool that more closely resembled that seen in earlier life [120]. This suggests that IL-7 therapy could enhance and broaden immune responses particularly among individuals with limited naive T cells and diminished repertoire diversity, as it occurs after chemotherapy-induced lymphocyte depletion. In a phase I study, IL-7 administered to patients with incurable malignancy markedly increased peripheral CD3^+^, CD4^+^, and CD8^+^ T cells and transitional B cells, and also, induced in the bone marrow a marked polyclonal proliferation of pre-B cells [124]. IL-7 therapy has also enhanced T cell recovery in HIV-1-infected adults [125, 126].

HGF administered as a gene-therapy or as a recombinant protein has been tested in clinical trials for hepatitis [77], fulminant liver failure [122], coronary artery disease [127–130], ischemic cardiac disease [131], and critical limb ischemia [132, 133].

McKenna et al. [134] identified a naturally occurring heterodimeric self-assembling form of IL-7 with the beta chain of hepatocyte growth factor, called *pre-pro-B cell growth-stimulating factor (PPBSF)* (HGF) [135, 136]. HGF is a heterodimeric, pleiotropic cytokine regulating parenchymal cell growth, motility, and morphogenesis [137]. HGF contributes to the regulation of hematopoiesis in mouse fetal liver and adult bone marrow [138, 139] as well as lymphopoiesis [140, 141].

The IL-7/HGF*β* hybrid cytokine selectively induces proliferation and differentiation of pre-pro-B cells in rat BM, by upregulating the IL-7R*α* chain and c*μ* expression, to enable pro-B cells to respond to monomeric IL-7 [142]. Lai et al. fused Il-7 and HGF*β* via a peptide flexible linker [143]. This recombinant single-chain (sc) IL-7/HGF*β* protein, unlike IL-7, stimulates the proliferation of day 12 spleen colony-forming units (CFU-S12) and pre-pro-B cells, as well as common lymphoid progenitors (CLPs). Furthermore, cells from scIL-7/HGF*β*-stimulated cultures generated B-lineage cells in vivo more effectively than did those from cultures stimulated with IL-7. scIL-7/HGF*β*s exhibit the so-called “juxtacrine signaling” via engagement of *both* the IL-7 and HGF (c-Met) receptors. Administration of the IL-7/HGF*β* hybrid cytokine significantly enhances thymopoiesis after syngeneic or allogeneic bone marrow transplantation and promotes antitumor immunity [144].

### 6.3. Cyt107, Recombinant Human IL-7 (rhuIL-7)

Several companies have initiated clinical trials of IL-7-based molecules.

The Cytheris SA molecule, Cyt107, has been the subject of 18 clinical trials according to http://Clinicaltrials.gov. Trials have sought to boost immunity in (a) chronic virus infections (HIV, hepatitis B, and hepatitis C); (b) recipients of hemopoietic stem cell transplants (HSCT); (c) various cancers (metastatic breast, Ewing's sarcoma, glioma, metastatic prostate [combined with Provenge], metastatic melanoma or locally advanced or metastatic kidney cancer, rhabdomyosarcoma, and neuroblastoma); (d) sepsis; and (e) idiopathic CD4 lymphocytopenia. Unfortunately, Cytheris SA filed for bankruptcy in June 2013, and presently, the development of rhuI-L7 has been suspended. Commercial rights have been returned to the French ANRS (National Agency for AIDS Research).

Initial first-in-human safety trials showed that a single cycle of 3 weekly subcutaneous (sc) injections of recombinant human interleukin 7 (r-huIL-7) was safe. r-huIL-7 improved restoration of immune CD4 T cell in human immunodeficiency virus- (HIV-) infected patients (NCT01241643). Two phase II trials evaluated the effect of repeated cycles of r-hIL-7 (20 *μ*g/kg). r-hIL-7 was well tolerated with four grade 4 events that were not life-threatening [145]. INSPIRE II (NCT01190111) was a single-arm trial conducted in the United States and Canada, whereas INSPIRE III (EudraCT; number 2010-019773-15) was a dual arm trial with 3 : 1 randomization of r-hIL-7 versus control conducted in South Africa and Europe. Eligible participants had plasma HIV RNA levels < 50 copies/mL during antiretroviral therapy and with CD4 T cell counts between 101 and 400 cells/*μ*L. A repeat cycle was given when CD4 T cell counts fell to <550 cells/*μ*L. A total of 107 patients were treated and received 1 (*n* = 107), 2 (*n* = 74), 3 (*n* = 14), or 4 (*n* = 1) r-hIL-7 cycles during a median follow-up of 23 months. After the second cycle of treatment, anti-r-hIL-7 binding antibodies were observed in 82% and 77% of patients in INSPIRE 2 and 3, respectively (neutralizing antibodies in 38% and 37%), without affecting the CD4 T cell response. 50% of the patients spent >63% of their follow-up time with a CD4 T cell counts > 500 cells/*μ*L. In conclusion, repeated cycles of r-hIL-7 achieved sustained CD4 T cell restoration to >500 cells/*μ*L and were well-tolerated in the majority of study participants.

#### 6.3.1. Cyt107 in Sepsis

Sepsis is characterized by life-threatening organ dysfunction caused by dysregulated host responses to infection [146]. Sepsis continues to be the primary cause of death in the intensive care unit despite decades of research and dozens of clinical trials of various targeted antisepsis associated proinflammatory agents. Despite a state of “hyper-inflammation,” sepsis impairs adaptive antimicrobial immunity characterized by compromised T cell function leading to aberrant antigen presentation with increased susceptibility to nosocomial infections such as *Pseudomonas aeruginosa.* Shindo et al. [147] demonstrated that recombinant huIL-7 improves survival in a murine 2-hit model of sublethal cecal ligation and puncture followed by *Pseudomonas aeruginosa* pneumonia. rhuIL-7 increased survival from 56 to 92% with increased absolute numbers of immune effector cells in lung and spleen reduced sepsis-induced loss of lung innate lymphoid cells (ILCs). Translation of this work to humans is in progress. A multicenter, randomized, double-blinded, placebo-controlled study of two dosing frequencies of CYT107 to restore absolute lymphocyte counts in sepsis patients [IRIS-7B (Immune Reconstitution of Immunosuppressed Sepsis patients)] is underway (http://Clinicaltrials.gov: NCT02640807). To allow a common statistical analysis of the primary end points and analysis of the enrolled patient population, a parallel study will be performed in France.

#### 6.3.2. Cyt107 in Idiopathic CD4 Lymphopenia

Idiopathic CD4 lymphopenia (ICL) is a rare syndrome defined by low CD4 T cell counts (<300/*μ*L) without evidence of HIV infection or other known cause of immunodeficiency. ICL suffer opportunistic infections with no established treatments. Sheikh et al. [148] initiated an open-label phase 1/2A dose-escalation trial of 3 subcutaneous doses of rhuIL-7 weekly in patients with ICL at risk of disease progression (#NCT00839436). The number and function of circulating CD4 and CD8 T cells and tissue-resident CD3 T cells in the gut mucosa and bone marrow was increased. Overall, recombinant huIL-7 was well tolerated; however, one patient developed systemic lupus erythematosus while on study and another experienced a hypersensitivity reaction with subsequent development of nonneutralizing anti-IL-7 antibodies.

#### 6.3.3. Cyt107 in Cancer

Cyt-107 has been evaluated in patients suffering from cancer. Most of this work has not been reported in the medical literature. The Fred Hutchinson Cancer Research Center with the National Cancer Institute (NCI) Cancer Immunotherapy Trials Network [http://ClinicalTrials.gov; NCT01881867] is the primary sponsor of a multicenter randomized phase II trial to evaluate glycosylated recombinant human interleukin-7 (CYT107) after vaccine therapy in patients with metastatic hormone-resistant prostate cancer.

### 6.4. IL-7 Fc Fusion Protein: GX-I7

GX-I7 is an immunoglobulin fusion protein recombining human IL-7 with a hybrid Fc (hyFc) being developed by Genexine, a Korean company, with USA subsidiary Neoimmune Tech Inc. based in Rockville, MD. hyFc is composed of the hinge-CH2 region of immunoglobulin D (IgD) and the CH2-CH3 region of immunoglobulin G4 (IgG4). This molecule is expected to have markedly reduced Fc receptor binding while maintaining FcRn binding and the characteristic long plasma half-life of an antibody (perhaps 2-3 weeks). A randomized, double-blind, placebo-controlled, single-ascending dose study designed to assess the safety, tolerability, pharmacokinetics, and pharmacodynamics of GX-I7 in healthy volunteers is presently recruiting patients (http://ClinicalTrials.gov; NCT02860715).

Published preclinical studies support the development of GX-I7, (a) as intranasal pretreatment for generating protective immunity against influenza infection [149], (b) as an adjuvant for DNA vaccines [150] and HCV [151], and (c) as an adjuvant for various T cell therapies [152].

### 6.5. Gene Therapy Employing IL-7

Numerous groups are utilizing transgenically delivered IL-7 or its receptor to augment antitumor immunity. Regulatory T cells (Tregs) limit the antitumor effects of tumor-specific CTLs [153–158]. Early work to increase in vivo immunostimulation of adoptively transferred T cells utilized IL-2 [159]. Although IL-2 is a potent T cell growth factor, it is not selective for effector T cell subsets and can augment the expansion and inhibitory activity of Tregs [160]. In contrast, IL-7 augments expansion of naive and memory T cells without activity on Tregs that lack the IL-7R*α* [114, 123, 161]. Furthermore, in early clinical trials, recombinant IL-7 expanded naive and central-memory T cell subsets but not Tregs [123, 124]. Vera et al. [162] reported that IL-7 cannot support the in vivo expansion of adoptively transferred chimaeric antigen receptor- (CAR-) redirected CTLs because these effector-memory T cells, like Tregs, also lack IL-7R*α*. To address this deficiency, Perna et al. [163] created GD2-specific CAR/CTLs bearing a functional transgenic IL7R*α* and showed that IL-7, in contrast to IL-2, can support the proliferation and antitumor activity of these cells both in vitro and in vivo in the presence of functional Tregs.

## 7. Conclusion: The Yin and Yang of IL-7 in Aging

The notion that low IL-7R expression levels are beneficial for reaching healthy old age corresponds with previous observations that patients suffering from autoimmune disease express increased levels of the IL-7 receptor/ligand complex genes [101, 102, 104] and that antagonizing IL-7 or the IL-7R may offer possible treatments [95, 101]. However, the results of the Leiden Longevity Study found that gene expression levels of IL-7R decrease with chronological age. On the other hand, the Leiden study also found that higher levels of IL-7R correlate with reduced 10-year mortality and that effect was pronounced in the nonagenarian population in which individuals at the high end of the overall lower IL-7R expression lived longer. To optimize health and lifespan, it may be useful to “thread the needle,” lowering IL-7R enough to preserve peripheral T cells and help maintain low mTOR levels, while maintaining enough to maintain immune function. Transient modulation of IL-7R is one potentially effective strategy to reach this goal. Another possible conclusion is “correlation is not causation” and that the genes of IL-7/IL-7R complex are only part of the answer.

The remarkable plasticity of the adaptive immune system over many decades is a testament to several intrinsic features of its design. Despite attacks on its integrity from multiple angles, the size and diversity of the naive lymphocyte repertoire is maintained well into the 9th decade of life. While IL-7 is a necessary contributor to this “lympho-homeostasis” and its action is *required* for successful aging, wholesale augmentation of IL-7 above “normal” levels may disrupt this delicate balance. Numerous animal and several human studies suggest much promise remains for the utilization of IL-7 as a specific “immune tonic” or adjuvant. To this end, we look forward to the next generation of improved IL-7-based therapeutics.
